# A Single-Domain VNAR Nanobody Binds with High-Affinity and Selectivity to the Heparin Pentasaccharide Fondaparinux

**DOI:** 10.3390/ijms26094045

**Published:** 2025-04-24

**Authors:** Martha Gschwandtner, Rupert Derler, Elisa Talker, Christina Trojacher, Nina Gubensäk, Walter Becker, Tanja Gerlza, Zangger Klaus, Pawel Stocki, Frank S. Walsh, Julia Lynn Rutkowski, Andreas Kungl

**Affiliations:** 1 Institute of Pharmaceutical Sciences, Karl-Franzens-University Graz, Universitätsplatz 1, A-8010 Graz, Austriaelisa.talker@uni-graz.at (E.T.); christina.trojacher@uni-graz.at (C.T.); tanja.gerlza@uni-graz.at (T.G.); 2 Institute of Chemistry, Karl-Franzens-University Graz, Heinrichstraße 28, A-8010 Graz, Austria; nina.gubensaek@uni-graz.at (N.G.); walter.becker@uni-graz.at (W.B.); klaus.zangger@uni-graz.at (Z.K.); 3 Ossianix, Inc., Stevenage Bioscience Catalyst, Gunnels Wood Rd, Stevenage, Herts SG1 2FX, UK; pawel@ossianix.com (P.S.); walsh@ossianix.com (F.S.W.); rutkowski@ossianix.com (J.L.R.); 4 Antagonis Biotherapeutics GmbH, Strasserhofweg 77a, A-8045 Graz, Austria

**Keywords:** VNAR, fondaparinux, chemokines, Glycosaminoglycans, phage display

## Abstract

Glycosaminoglycans (GAGs) are key ligands for proteins involved in physiological and pathological processes. Specific GAG-binding patterns are rarely identified, with the heparin pentasaccharide as an Antithrombin-III ligand being the best characterized. Generating glycan-specific antibodies is difficult due to their size, pattern dispersion, and flexibility. Single-domain variable new antigen receptors (VNAR nanobodies) from nurse sharks are highly soluble, stable, and versatile. Their unique properties suggest advantages over conventional antibodies, particularly for challenging biotherapeutic targets. Here we have used VNAR semi-synthetic phage libraries to select high-affinity fondaparinux-binding VNARs that did not show cross-reactivity with other GAG species. Competition ELISA and surface plasmon resonance identified a single fondaparinux-selective VNAR clone. This VNAR exhibited an extraordinarily stable protein fold: the beta-strands are stabilized by a robust hydrophobic network, as revealed by heteronuclear NMR. Docking fondaparinux to the VNAR structure revealed a large contact surface area between the CDR3 loop of the antibody and the glycan. Fusing the VNAR with a human Fc domain resulted in a stable product with a high affinity for fondaparinux (Kd = 9.3 × 10^−8^ M) that could efficiently discriminate between fondaparinux and other glycosaminoglycans. This novel glycan-targeting screening technology represents a promising therapeutic strategy for addressing GAG-related diseases.

## 1. Introduction

Glycosaminoglycans (GAGs) are a large class of linear, unbranched, and negatively charged polysaccharides with molecular weights in the range of 10–100 kDa. GAGs consist of repeating disaccharide units composed of a uronic acid and an amino sugar, which are post-synthetically sulfated at various positions along the sugar backbone by site-specific sulfotransferases, derivatizing hydroxyl groups as well as the amino group, the latter of which may also undergo enzymatic acetylation [1,2]. Members of the GAG family include heparan sulfate, chondroitin sulfate, dermatan sulfate, keratan sulfate, and non-sulfated hyaluronic acid [3]. Synthesis of sulfated GAG chains occurs in the Golgi apparatus, initiated by O-glycosylation of the core protein, except for keratan sulfate, which may undergo either N- or O-glycosylation [4]. GAGs exert their biological and pathological functions by interacting with proteins, which is driven mainly by electrostatic interactions but is supported by site-targeting, less far-ranging van der Waals, and hydrogen bonding interactions [5,6]. These interactions play a crucial role in various biological and pathological processes, including cell signaling, angiogenesis, anticoagulation, tumor progression, and axonal growth. While typical linear consensus sequences found in many GAG-binding proteins are XBBXBX and XBBBXXBX (where B denotes a basic amino acid), the overall fold of proteins can constitute a three-dimensional GAG-binding domain, in addition to single linear binding sequences [7].

**Figure 1:**
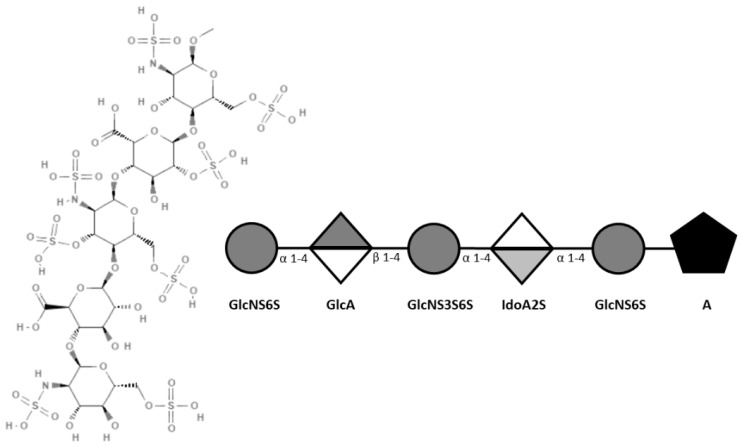
Fondaparinux structure (Pubchem 5282448). ATII binding motif Glc-NAc6S-GlcA-GlcNS3S6S-IdoA2S-GlcNS6S.

Deciphering the structural prerequisites of GAGs that govern their binding specificity with different proteins holds significant promise for developing new therapeutic interventions targeting diseases associated with pathological GAG expression patterns. The most prominent example of a specific, high-affinity protein-binding GAG sequence is the heparin (HP) pentasaccharide GlcNAc6S-GlcA-GlcNS3S6S-IdoA2S-GlcNS6S (see Figure 1), commercially known as fondaparinux (Arixtra^®®^). Fondaparinux sodium is methyl-O-2-deoxy-6-O-sulfo-2-(sulfoamino)-α-D-glucopyranosyl-(1→4)-O-β-D-glucopyranuronosyl-(1→4)-O-2-deoxy-3,6-di-O-sulfo-2-(sulfoamino)-α-D-glucopyranosyl-(1→4)-O-2-Osulfo-α-L-idopyranuronosyl-(1→4)-2-deoxy-6-O-sulfo-2-(sulfoamino)-α-D-glucopyranoside, deca sodium salt. Fondaparinux sodium binds to Antithrombin-III (AT-III) with a Kd ranging from 20 to 60 nM, thereby enhancing its anticoagulant function through increased inhibitory effects on thrombin [8,9]. While no other comparable high-affinity protein-specific GAG sequences have been identified to date, preferences for binding have been elucidated for certain GAG structures over others using libraries of differentially sulfated GAG fragments and other analytical techniques. Understanding the intricacies of GAGs and their impact on structure-function relationships is pivotal for understanding the GAG interactome and predicting high-affinity binding partners [10,11,12,13].

Currently, most FDA-approved therapeutic antibodies are full-size IgG antibodies with a size of about 150 kDa. However, the inherent limitations of full-size antibodies, including suboptimal tissue penetration and difficulty binding to certain molecular surface regions inaccessible to large proteins, have prompted the exploration of alternative antibody scaffolds [14]. Single-domain antibodies (sdAbs) present a promising solution to these challenges, offering attributes such as compact size, improved tissue penetration, monomeric behavior, high solubility, robust stability, and heightened specificity. Single-domain scaffolds, originating from diverse proteins and species, serve as versatile platforms for therapeutic development [15,16,17]. Engineered into multivalent formats, sdAbs have demonstrated their adaptability, giving rise to bivalent or bispecific nanobodies based on variable new antigen receptors (VNARs), heavy-chain variable domains (VHHs), and heavy chains (VHs) [18,19,20]. The immunoglobulin new antigen receptor from the nurse shark Ginglymostoma cirratum, referred to as ‘IgNAR’, appears as a disulfide-bonded dimer of two protein chains. It is an H (heavy)-chain homodimer with five constant domains, each chain consisting of one variable domain (VNAR’), in contrast to human IgGs, which consist of two heavy and two light chains covalently linked via disulfide bonds [21,22,23,24,25]. The variable domains recognize antigens with only a single immunoglobulin domain, similar to camelid VHH domains.

The distinctive features of VNAR nanobodies include a primary binding surface formed by a relatively short CDR1 loop and a longer CDR3 loop, in contrast to human VHs, in which the binding site is shaped by CDR1-3 [26]. The hypervariable loop 2 resides at the base of the VNAR structure, while the hypervariable loop 4 is situated between HV2 and CDR3 [27]. Notably, in both types, the extended CDR3 loop facilitates access to cleft-like epitopes, underscoring the unique structural characteristics of VNAR nanobodies compared to traditional antibodies [25].

Toin van Kuppevelt’s research on glycosaminoglycan (GAG) antibodies has significantly contributed to our understanding of the role of GAGs in various physiological and pathological processes [28,29,30]. His group developed and characterized antibodies specific to different types and/or sulfation patterns of GAGs. These antibodies serve as valuable tools for studying the distribution, structure, and function of specific GAG patterns in various biological organs and tissues. By targeting specific GAG epitopes, researchers can elucidate the roles of these complex molecules in health and disease. By utilizing GAG-directed antibodies, researchers can investigate how alterations in GAG expression and structure contribute to disease progression and identify potential therapeutic targets. The antibodies that were raised against specific GAGs were selected using a human semi-synthetic phage library (containing >108 different clones), with randomized complementary region 3 (CDR3) regions ranging between 4 and 12 amino acids. This CDR3 exhibits the highest conformational variability and flexibility and has a major influence on binding properties [28,31,32,33,34]. The van Kuppevelt group engineered a large panel of different antibodies against GAGs in the past decades, which depict different epitope specificities and modifications involved in binding. These single-chain variable fragments consist of a fixed VL chain that is linked to a variable VH chain [35]. None of these antibodies was considered for therapeutic use.

We explored this topic further by screening highly diverse type 2 and type 4 semi-synthetic VNAR phage libraries [36] with a single highly defined (since synthetic) GAG antigen. The CDR3 regions of VNARs have superior characteristics due to their structural diversity and adaptability, which enable VNARs to bind to a wide range of target antigens with high specificity. VNARs’ intricate CDR3 loop can span up to 34 amino acids, far exceeding the typical 12 amino acids in mouse or human VH [37,38]. This unique feature, coupled with their compact size, enables VNARs to target epitopes that are inaccessible to conventional IgG antibodies [24,39]. The present study aimed to isolate a VNAR antibody that recognizes for the first time a single GAG motif with high affinity. To accomplish this objective, fondaparinux pentasaccharide was employed as an antigen in a VNAR phage display library selection process, aiming to unravel glycan-binding motifs that exhibit a strong selectivity for the designated GAG molecule and are characterized by a high affinity.

VNARs have already been successfully used to generate binding molecules against several targets using different phage or ribosome display techniques [27,40,41,42]. Panning was performed using streptavidin-coated beads in a solution-based approach to enhance antigen accessibility compared to plate-oriented setups. To enable this selection, the antigen required biotinylation without altering its binding affinity. Rigorous exclusion of false-positive clones raised against biotin itself was imperative. Cross-reactive VNARs that recognized alternative GAG species were systematically excluded, yielding a final fondaparinux (FP)-binding VNAR. Subsequently, this VNAR was reconstituted into an Fc backbone for a comprehensive analysis of its binding and functional characteristics. Our findings revealed a functional VNAR antibody exhibiting exceptional specificity for fondaparinux, with only minimal cross-interaction with other GAG species.

## 2. Results

### 2.1. Characterization of Biotinylated Fondaparinux (b-FP)Subsection

**Figure 2:**
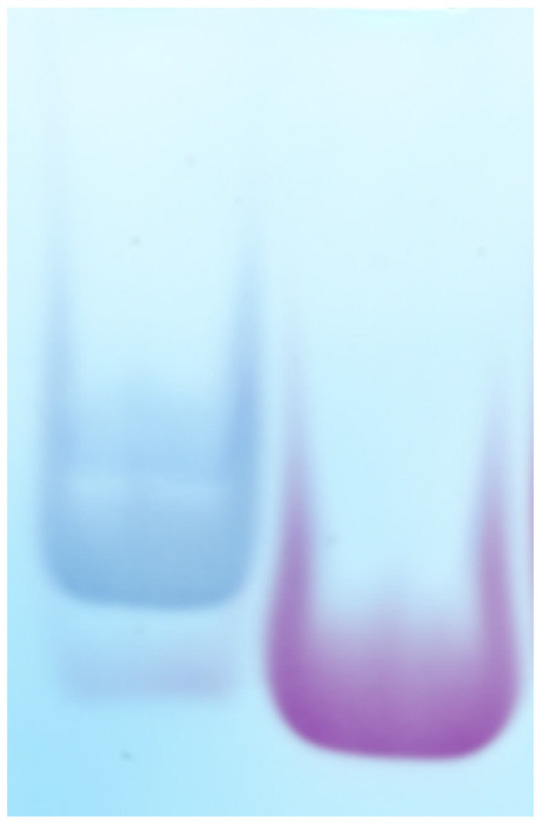
Oligosaccharide PAGE of Biotinylated FP (left lane) versus unbiotinylated FP (right lane) stained with Azur A.

Utilizing Oligosaccharide-Polyacrylamide Gel Electrophoresis (PAGE), we showed that approximately 90% of fondaparinux underwent successful biotinylation (Figure 2). Isothermal Fluorescence Titration (IFT) experiments showed that Antithrombin-III (AT-III), the natural fondaparinux target protein, exhibited almost identical Kd values for b-FP (42 nM) compared to non-biotinylated FP (49 nM). This indicates that the binding surface of the FP penta-saccharide remained intact after biotinylation, which is important for subsequent phage display screening.

Detailed Mass Spectrometry (MS) analysis revealed a predominant peak corresponding to singly biotinylated fondaparinux (FP). Additionally, several peaks exhibiting different charge states and distinct Na+/K+ adduct peaks were observed. Peaks corresponding to unbiotinylated or double-biotinylated FP were also observed, albeit with minor intensities.

### 2.2. Anti-Fondaparinux VNARs

Fourteen distinct clones were successfully isolated through two phage display selection rounds, each demonstrating selective binding to biotinylated fondaparinux (b-FP) while exhibiting no discernible affinity for other glycosaminoglycans (GAGs), with the exception of low-molecular-weight heparin (LMWH) heparin, which contains the FP motif to a limited extent.

Two main VNAR families corresponding to the type 4 and type 2 isoforms were identified. In selection round Series 1 (see Table 1), seven unique VNARs featuring a consensus NVY motif in the CDR3, along with a combined total of 4–5 basic residues distributed across CDR1 and CDR3, were identified. Sequencing the DNA of 90 clones in selection round Series 2 (Table 2) revealed five VNARs sharing a CDR3 PRXXXXSCQGSSRR consensus motif coupled to an identical CDR1 region. Notably, Fonda054-D09 diverged as a distinct clone, characterized by a basic arginine cluster in CDR3 and a unique CDR1. Fonda054-A02 exhibited similarities to the clones in Series 1, featuring an asparagine at position 1, tyrosine at position 4, and serine at position 7 within the CDR3 region.

While the existing literature proposes GAG-binding motifs for proteins, such as XBBXBX or XBBBXXBX, where X represents any amino acid and B denotes a basic amino acid [43], it is crucial to note that some well-known GAG-binding proteins, including the CDR3 regions of heparan sulfate (HS) binding antibodies (e.g., SRKTRKPFMRK and HAPLRNTRTNT), do not conform to such sequences. Consequently, relying on these sequences as definitive indicators of GAG-binding potential is of limited use [44,45,46].

In light of the binding analysis conducted on the resulting VNAR nanobody clones, a discernible amino acid pattern emerged that enhanced the specificity of binding to fondaparinux. Notably, a substitution pattern was identified for VNARs that exhibited specificity toward fondaparinux. Specifically, the substitution of histidine (H) at position 1 and glycine (G) at position 7 with other basic amino acids like lysine or arginine was found to enhance binding. Additionally, the substitution of arginine (R) at position 5 with hydrophobic amino acids (F, I, L, M, V, and W) and the replacement of aspartic acid (D) at position 8 with polar amino acids were effective in strengthening FP binding. Conversely, weaker binding to fondaparinux was observed when arginine was replaced with a non-basic amino acid at positions 1, 5, and 7 in the amino acid sequence. These findings provide valuable insights into the specific amino acid residues required for robust and selective binding to fondaparinux.

### 2.3. Structural Analysis and Molecular Modelling

Based on the monoclonal phage ELISA results, Fonda054-D09, a type 2 VNAR, was selected as a specific fondaparinux VNAR for further structural studies. NMR studies were performed to resolve the structure of this novel VNAR nanobody monomer without Fc fusion (see Table 3). For the structure calculation, only the core domain (residues 24 to 134) was analyzed, as residues 1–23 and 135–140 did not form a secondary structure. The structural elucidation of VNAR nanobody reveals an immunoglobulin fold with a typical beta-sandwich framework consisting of nine beta-strands. On one side of the structure, the strands are connected by short turns; on the other side, long loops containing disulfide bridges form the hypervariable region, which, in this case, is the heparin-binding site. The heparin-binding region is formed by two loop domains (residues 110–124 and 49–56) connected by a thioester bond (C52–C114). A second disulfide bridge is formed between C110 and C119. The heparin-binding region is not visible in the NMR spectra, likely due to conformational exchange on an intermediate timescale.

**Figure 3:**
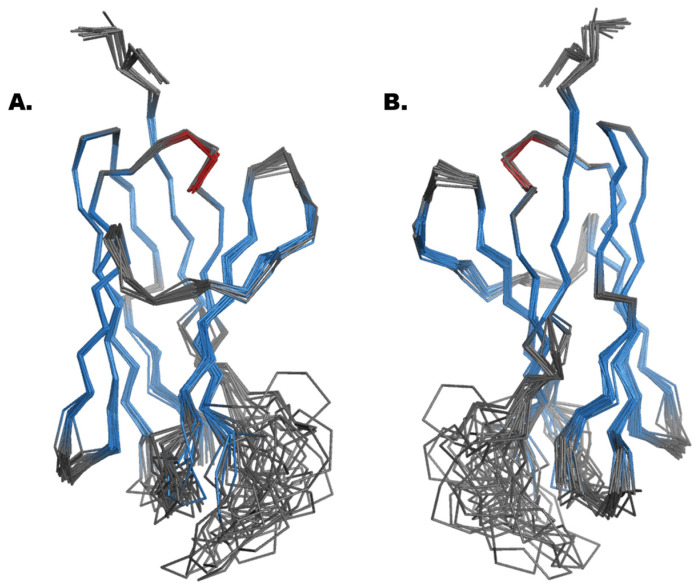
Alignment of 20 Fonda054-D09 VNAR structures as a ribbon model (beta sheets are displayed in blue color; loop regions are displayed in grey). The heparin-binding site is located in the flexible loop region (residues 110–124 and 49–56; see Figure 4 below). (**A**) front view. (**B**) 180° turn view.

The VNAR showed an extraordinarily stable fold (see Figure 3), and the beta-strands were stabilized by a strong hydrophobic network. Additionally, a disulfide bridge connects and stabilizes the protein fold. A bridge is formed between C45 and C106 and thus connects beta-strand 3 with beta-strand 8. A closer look inside the hydrophobic core provides evidence for π-π stacking forces among residues Phe89 and Trp59, improving the stability of the beta-sandwich framework.

**Figure 4:**
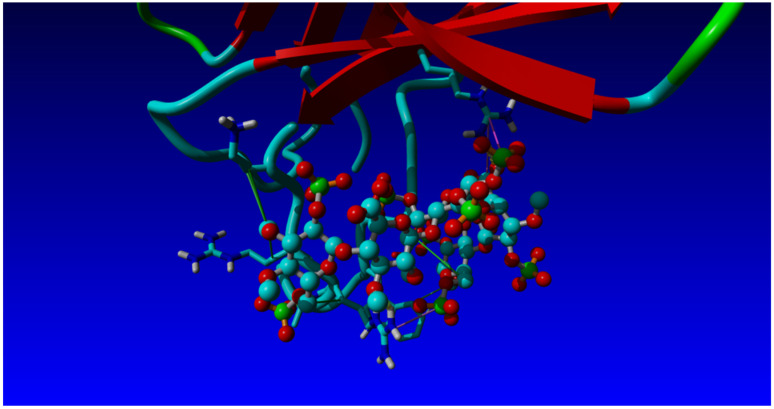
View of the CDR1 and CDR3 regions of the anti-fondaparinux VNAR in complex with fondaparinux. Displayed in stick mode are the amino acids in the CDR3 loop responsible for fondaparinux binding; displayed in ball-and-stick mode is the fondaparinux molecule (colored in turquoise is carbon, red is oxygen, blue is nitrogen, and green is sulfur).

Based on the NMR structure of Fonda054-D09, the structure of VNAR in complex with heparin fondaparinux was generated by molecular modelling [47]. For this purpose, a fondaparinux pentasaccharide structure was obtained from the 1azx.pdb file [48] (PDB DOI: https://doi.org/10.2210/pdb1AZX/pdb; deposited on 23 December 1997), i.e., from the complex structure of Antithrombin-III and fondaparinux, and was subsequently subjected to rigorous energy minimization and molecular dynamics relaxation. The resulting conformationally unconstrained pentasaccharide was used as a ligand in docking studies of Fonda054-D09 using AutoDock contained in the YASARA molecular modelling program package (see Materials and Methods, Section 4). Fourteen complex structures with similar binding energies were identified, and the complex with the largest contact surface area between VNAR and fondaparinux is shown in Figure 4. Eight of the eleven amino acids in the CDR3 region were in contact with fondaparinux, and one was from the CDR1 domain (Figure 4). Interestingly, the 3-*O* sulfated middle 3-*O*,6-*O*, N-*S* glucosamine moiety of fondaparinux, which is the Antithrombin-III specific group of this glycan, is strongly coordinated by Arg82, which lies outside the CDR3 domain of the VNAR: together with the other amino acids within the CDR3 domain, this interaction pattern is suggested to make the Fonda054-D09 VNAR specific for fondaparinux.

Particularly, two more hydrogen bonds have been identified that energetically stabilize the complex strongly: Arg89 coordinates via its backbone -NH group as well as with its sidechain -NH3^+^ group to the neighboring iduronic acid and 6-O,N-S glucosamine moieties.

### 2.4. Fc Fusions and Fondaparinux Binding

In the next step, Fonda054-D09 was fused to a human Fc domain, where the VNAR is in a bivalent format, in order to determine how valency and affinity are affected by Fc fusion. Both affinity and specificity were investigated using SPR experiments. In order to suppress non-specific electrostatic interactions and focus on specific interactions only, 400 mM NaCl was added to the binding buffer. This may result in a lower relative affinity but strongly hints at selectivity.

**Figure 5:**
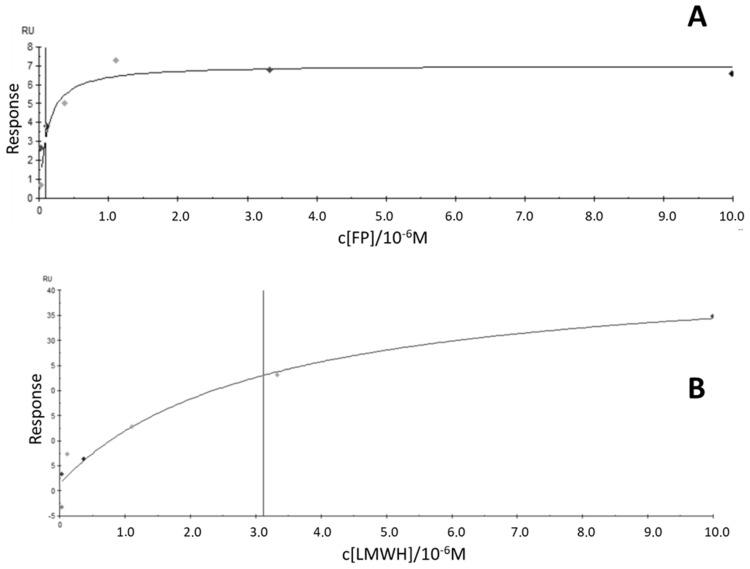
Bimolecular binding curves obtained by SPR using biotin-immobilized fondaparinux (FP, (**A**)) and low-molecular-weight heparin (LMWH, (**B**)) and Fc-Fonda054-D09 as interaction partners. The K_d_ values were obtained using the standard Biacore X100 analysis program.

The bivalent Fc-Fonda054-D09 bound specifically to fondaparinux (FP) with a Kd value of 9.3 × 10^−8^ M (see Figure 5) and showed no interaction with HS and CS. With low-molecular-weight heparin (LMWH), Fc-Fonda054-D09 interacted with a Kd value only in the µM range (Kd = 3.1 × 10^−6^ M). For comparison, two other Fc fusion constructs were cloned, expressed, and subjected to SPR experiments: Fc-Fonda054-G10 did not bind to FP at all and showed a µM binding affinity for LMWH but no binding to HS and CS. Fc-Fonda054-A01 did not display any binding to FP, but Kd values in the lower µM range were observed for LMWH and HS (no affinity for CS was detected).

## 3. Discussion

Glycosaminoglycans (GAGs) are highly negatively charged linear polysaccharides that serve as co-receptors for various proteins. These interactions are involved in multiple physiologic processes and a multitude of severe pathologic conditions. The expression and structural patterns of GAGs are subject to age-, extracellular signal-, and disease-related changes. Targeting specific GAG structures could also be a valuable diagnostic tool; however, it represents a novel therapeutic strategy to address GAG-related diseases.

Phage display screening of two VNAR libraries, type 2 and type 4, was performed to identify novel GAG-binding antibodies with the desired properties. VNAR nanobodies are highly soluble, very stable, and have exceptionally versatile binding domains. Due to their deletion of the CDR2 region, VNARs are the smallest natural single-domain antibodies, ranging around 12 kDa, and smaller than VHH or Vh domains [49]. Thus, VNAR domains consist of only two complementarity-determining regions: CDR1 and CDR3. Despite having a reduced number of antigen-binding loops, they can exhibit high affinities in the low nanomolar range for a large array of antigens [50]. The primary source of diversity in the VNAR repertoire is predominantly concentrated in the CDR3 region [51]. The extended and structurally complex CDR3 length in VNARs can be up to 34 amino acids and surpasses that of mouse or human VH, with a maximum of 12 amino acids [37,38]. The combination of small size and long CDR3 loops, in conjunction with intermolecular support, allows VNARs to reach buried epitopes, which could be inaccessible for IgG with their flat or concave antigen-binding sites and conveys a high penetration capability [24,39]. Polar and charged amino acids are more common at solvent-exposed surfaces, making them highly soluble [51]. VNARs compensate for their smaller antigen-binding surface area compared to conventional antibodies by having longer CDR3 loops, which, in combination with the antibody’s β-strand framework, create diverse antigen-binding surfaces that can recognize a wide range of target antigens. VNARs can undergo rapid somatic hypermutation in response to antigens, enhancing their affinity for the target [22]. These mutation-prone regions are designated HV2 and HV4 [52]. Due to the urea content in shark blood for osmotic regulation, VNARs tend to be highly resistant to chemical or enzymatic degradation and also exhibit higher pH and thermostability, which can be accomplished by the presence of disulfide bridges and hydrogen bonds [39,53].

Despite their prevalence on the surfaces of cells and virus particles, carbohydrates typically do not trigger immune responses in the same way as peptide antigens. Immune responses to carbohydrate antigens typically occur when these structures consist of monosaccharides and oligosaccharides that are foreign to the host glycan structures [54]. While human glycoconjugates exhibit a vast array of structural diversity, the core of human glycosylation is built upon a relatively limited repertoire of just ten monosaccharides. In contrast, bacterial glycosylation is characterized by a far more extensive range, encompassing over a hundred distinct monosaccharides. Naturally occurring carbohydrate-binding antibodies that identify bacterial, fungal, and other microbial carbohydrates play a crucial role in averting systemic infections and preserving the equilibrium of the microbiome [55]. Furthermore, beyond the composition of monosaccharides, the conformation and, consequently, the antigenic properties of carbohydrates are primarily determined by the specific glycosidic linkages that connect these monosaccharides [56]. Designing antibodies against these carbohydrate structures is thus a challenge, as carbohydrates exhibit different conformations, and the antibody should ideally differentiate between these conformations to target disease-specific antigens only and thus limit cross-reactivity. Another limiting factor is the inaccessibility of carbohydrates, which are embedded in a dense network. VNARs can overcome these limitations due to their high penetrability efficiency. Knowledge of these well-defined carbohydrate antigen structures involved in disease is still scarce. In this study, we designed a VNAR nanobody that targets a well-defined pentasaccharide, known as the Antithrombin-III binding site. This “heparin pentasaccharide sequence”, is a specific structural motif found within the heparin molecule. This sequence consists of five sugar units, and its specific arrangement is crucial for the interaction between heparin and antithrombin, a natural anticoagulant in the blood. The pentasaccharide recognition site is primarily responsible for the anticoagulant activity of heparin and heparin-like drugs, such as fondaparinux (Arixtra^®®^) [57,58]. This sequence binds to Antithrombin-III, inducing a conformational change in the protein. As a result, Antithrombin-III becomes a more effective inhibitor of coagulation enzymes, especially factor Xa and thrombin. This inhibition of coagulation enzymes prevents the formation of blood clots [59].

We isolated multiple VNAR hits against a specific pentasaccharide that is known to bind Antithrombin-III with high affinity and analyzed its binding potency compared to other glycosaminoglycans. Fondaparinux served as the antigen in a phage display setup aimed at identifying high-affinity and selective binders for fondaparinux itself, excluding heparin, HS, or CS. To achieve this, fondaparinux was biotinylated, immobilized on streptavidin beads, and subjected to screening against a semi-synthetic VNAR library. The identification of selective fondaparinux binders was performed using ELISAs, SPR, and a functionality assay. The lead clones were subsequently purified and characterized for their purity, affinity, and selectivity across different GAG classes. These clones were then reformatted into Fc-constructs. To ensure the specificity of binding, their binding characteristics were further evaluated, ruling out any non-specific contributions attributed to the His-tag or biotin on the antigen, both of which were necessary for collecting fondaparinux binders. One of the isolated VNAR nanobodies showed little cross-reactivity with heparin, which also incorporates this pentasaccharide sequence as a natural Antithrombin-III binder, but no interaction was observed with HS or CS. It is notable that the FP binders are high-affinity and in the nM range with large CDR3 regions straight from the libraries and do not require affinity maturation at this stage. This is in contrast to most antibody screens against carbohydrate antigens, where, in general, the antibodies to GAGs have modest to low micromolar affinity with small CDR3 regions [53,56].

Apart from this example, the specific GAG structures responsible for the selective binding of different proteins are unknown, even though binding preferences for some prominent interaction partners, such as CXCL4 ((UA-GalNAc4S6S)2), CXCL8 ((IdoA2S-GlcNS6S)n), CXCL10 ((UA-GalNAc4S6S)2), CCL5 (UA2S-GlcNS6S-IdoA2S-GlcNS), and VEGF165 have been demonstrated [10,12,60,61,62,63,64]. Especially Tumor-Associated Carbohydrate Antigens (TACA) and glycans, which are found in many malignant cells, have high potency in the specific treatment of cancer [65]. For example, anti-GD2 monoclonal antibodies have been developed and approved for the treatment of neuroblastoma. GD2 belongs to the acidic glycosphingolipids and is overexpressed in a variety of cancers [66]. To target specific tumors, antibodies to the tumor-associated ganglioside GD2 have been isolated, and maturation technologies have been used to attain high affinity and specificity, which have been successfully used in clinical studies [54,67]. Sterner et al. (2016) elucidated the challenges inherent in procuring high-quality antibodies targeting glycans, revealing discrepancies in antibody performance across various glycan antigens [54]. They underscored the nuanced interplay of factors, such as self-versus, non-self-recognition and experimental parameters, which only partially account for the observed variability in antibody efficacy. They conducted experiments involving glycan microarrays to assess the binding affinity of two antibodies targeting GD2. Remarkably, these antibodies exhibited substantial selectivity for GD2, demonstrating binding strengths 250–1000 times weaker than those of GQ2 and GT2. Moreover, they displayed negligible interactions with numerous other potential antigens, underscoring their specificity for GD2 [67]. However, this is a relatively rare example, and there is a need for better technologies. Mammalian cell surfaces are encompassed by a dense layer of carbohydrates covalently linked to proteins, lipids, and ceramides. In the context of cancer, the intricacies of glycosylation, governed by glycosyltransferases and glycosidases, play a pivotal role in the development of malignancy. Therefore, it is crucial to focus on understanding glycosylation processes and the changes in the glycocalyx during disease progression [68,69].

Due to the alteration of the proteoglycan and, thus, glycosaminoglycan pattern during specific diseases, the generation of this new class of VNAR nanobodies is a promising new therapy to target these complex carbohydrates and ultimately develop unique therapeutics [70]. Due to their unique structural features, VNARs can be raised against cryptic antigens and engineered to have high affinity and specificity for their target antigens, making them suitable for therapeutic use, enabling precise and effective targeting of disease markers or pathogens, and limiting off-target effects. Due to their rather simple engineering and modification, they can even be formatted in a bi- or multispecific format, enhancing their therapeutic potential. Proteoglycans are part of a dense and crowded network, which makes them rather inaccessible to conventional, huge immunoglobulins. Due to their compact structure VNARs are able to penetrate the extracellular matrix and reach proteoglycan-rich areas. In summary, the distinctive attributes of VNARs, including their diminutive stature, robust stability, and remarkable adaptability, render them promising candidates for therapeutic interventions targeting proteoglycan patterns. Their inherent qualities herald a substantial reservoir of potential for forthcoming advancements in the realm of medical applications.

## 4. Materials and Methods

### 4.1. Materials

Phosphate-buffered saline (PBS) pH 7.2 contains 10 mM phosphate buffer and 137 mM NaCl. Fondaparinux (FP) was obtained from Merck (Darmstadt, Germany). All chemicals, unless stated otherwise, were purchased from Merck (Darmstadt, Germany) unless stated otherwise.

### 4.2. Antigen Preparation

#### 4.2.1. Biotinylation of Fondaparinux

**Figure 6:**
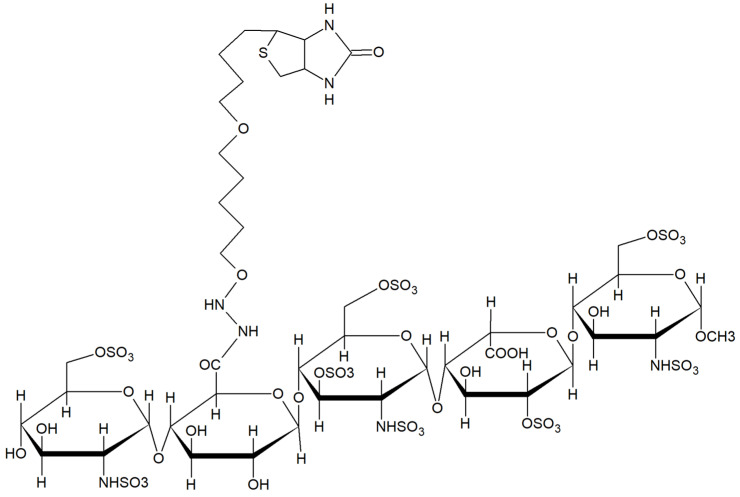
Schematic representation of carboxy biotinylation of fondaparinux. GAGs (FP, HS, CS, LMW Heparin) were biotinylated using EZ-Link^TM^ Hydrazide Biotin and EDC.

Fondaparinux (FP) was chosen as a model antigen to determine whether it was possible to isolate unique high-affinity glycan-binding VNAR nanobodies. Before phage display screening, fondaparinux was required to be coupled to magnetic beads; therefore, it was carboxy-biotinylated for further coupling onto streptavidin-coated beads. FP is a chemically synthesized AT-III-binding pentasaccharide of heparin (Figure 6). A homogenous antigen is essential for antibody screening, as it largely increases the chances of finding specific, high-affinity binders. FP was diluted in 0.1 M MES buffer pH 5.0 with 1.25 mM EZ-LinkTM Hydrazide Biotin (Thermo Fischer, Hillsboro, OR 97124, USA) and 6.5 mM EDC ((1-ethyl-3-(3-dimethylaminopropyl)carbodiimide hydrochloride; Thermo Fischer). After 2.5 h of incubation at RT, FP was dialyzed against HPLC-grade H_2_O for 48 h under gentle stirring using 100–500 Da MW cutoff Float-a-Lyzers (Thermo Fischer; Hillsboro, OR 97124, USA). To check the cross-reactivity of VNARs with other GAG species, heparan sulfate (Celsus, Cincinnati, OH 45241-1569, USA), low-molecular-weight heparin (Iduron, Alderley Park, Cheshire, SK10 4TG, UK), chondroitin sulfate (Biosynth Ltd., Compton, Berkshire, RG20 6NE, UK), and dermatan sulfate (Celsus, Cincinnati, OH 45241-1569, USA) were biotinylated using the same protocol.

#### 4.2.2. MS Analysis

MS analysis was performed to examine the amount of biotinylation and the sample purity. Thus, the desalted, dialyzed solution was speedvac dried at 40 °C, and the residual was diluted in 20 μL of GAG MS buffer (H_2_O:MeOH: NH_4_OH/50:50:0.1). Ten microliters of each dilution was transferred to borosilicate emitters using loader tips and connected to the MS system. The samples were measured using the following parameters: spray voltage 1.1 keV; capillary voltage −33 V; tube voltage 15 V; capillary temperature 210 °C; scan area 150–1000 *m*/*z*; scan events 3 micro scans; injection time 100 ms; MS^2^ isolation width 1.5 Da; collision energy 31%; collision RF potential (Q) 0.25; collision time 30 ms. The spectra were recorded for 2 min. Z (charge) was determined by a zoom scan and in MS^2^ by the neutral loss of SO_3_.

#### 4.2.3. Oligosaccharide PAGE

The purity of the pentasaccharide was analyzed by Oligosaccharide PAGE using 20% polyacrylamide gels, followed by Azur A staining. The separating gel buffer consisted of 10 mM Tris-HCl and 1 mM EDTA at pH 7.3. Gels were vertically poured between 1 mm glass spacer plates, and 40 mM Tris-HCl and 1 mM EDTA, pH 8.0 were used as the running buffer. Five micrograms of GAG was mixed with sample buffer (10 mM Tris-HCl, 1 mM EDTA, pH 7.3, and 20% glycerol) and loaded onto the gels. Electrophoresis was performed for 100 min at a constant voltage of 100 V. After Azur A staining, the gels were scanned using a GS800 Calibrated Densitometer (Biorad Inc., Hercules, CA 94547, USA).

#### 4.2.4. Isothermal Fluorescence Titration (IFT)

Isothermal fluorescence titration measurements (IFT) were conducted to validate the sustained binding affinity between fondaparinux and Antithrombin-III (AT-III). This method, known for its high sensitivity and robustness, relies on the observation of a reduction in the fluorescence signal. This quenching phenomenon is attributed to the structural rearrangement of the protein after interaction with the ligand, where a higher binding affinity corresponds to a greater quenching effect. A crucial precondition for performing IFT measurements is the presence of tryptophan (Trp) residues within the protein as intrinsic chromophores. IFT measurements were carried out on a Jasco Spectrofluorometer (FP6500, Jasco, 2967-5, Ishikawa-machi, Hachioji, Tokyo 192-8537, Japan) at an excitation wavelength of 280 nm, with measurements of the emission over the range of 300–400 nm. Additional settings included slit widths of 5 nm for both excitation and emission, a temperature of 20 °C, and a scan speed of 500 nm/min. AT-III underwent a 30 min equilibration period before measurement, and increasing concentrations of FP or bFP were added to achieve a final concentration of 400 nM. Following each titration step, the solution was mixed and allowed to equilibrate for 1 min before recording the spectra. The area under the curve of the obtained spectra was used for subsequent data analysis. The binding isotherms were obtained by plotting the relative change in fluorescence intensity (ΔF/F0) against the concentration (C) of the added ligand. The mean and deviation of three independent measurements were calculated and subjected to further analysis using Origin 8.0 (OriginLab, Northampton, MA, USA) through a non-linear regression fit [71].

#### 4.2.5. Phage Display Selection Campaign

The genotype and phenotype are directly linked in phage display because the gene of interest is fused to a phage coat protein. Therefore, the phage displays the protein on its surface, allowing the selection of antigen-binding peptides/proteins [72].

We developed phage libraries that contained over 10^10^–10^11^ different VNAR clones created by randomization of the VNAR CDR3 regions. OSX3 is a semi-synthetic type 2 VNAR library with randomized VNAR CDR3 regions ranging from 11 to 18 amino acids, with a flexible position of the canonical cysteine in the CDR3 loop [73]. CDR3 randomization was achieved using an oligonucleotide-directed randomization approach called NNK-randomization [74]. OSX4 is a type 4 semi-synthetic library, which is unique because there is no cysteine in the CDR3 loop, rendering the CDR3 region quite flexible [75]. The DNA encoding the VNAR variants was batch-cloned into the phagemid vector pSEX81 as a fusion to the gene encoding the phage coat protein pIII. To select specific epitopes, FP was immobilized on magnetic beads.

A starter culture was prepared by inoculating a single colony of freshly streaked ER2738 *E. coli* from an Agar plate into 50 mL 2 × TY medium (16 g Tryptone, 10 g Yeast extract, 5 g NaCl), filling it up to 1 L with dH_2_O containing 5 µg/mL tetracycline, and shaking it overnight at 37 °C and 200 rpm. A one-time use, ventilated 1 L shake flask with lid was used, and the volume of cells necessary to reach a starting OD600 of 0.1 in 500 mL 2 × TY medium containing 5 µg/mL tetracycline was added. The cells were grown at 37 °C and 200 rpm shaking. OD600 was measured regularly until it reached 0.5.

Cells were infected with helper phages M13K07 (1 × 10^13^ CFU/mL), incubated for 30 min without shaking at 37 °C, and centrifuged at 2000× *g* for 15 min at 4 °C. The supernatant was discarded, and the medium was replaced with 2 × TY containing 100 µg/mL ampicillin, 50 µg/mL kanamycin, and 5 µg/mL tetracycline. The flasks were incubated overnight at 30 °C with shaking at 200 rpm.

For the precipitation of phages, the medium was centrifuged at 2000× *g* for 15 min at 4 °C.

For precipitating, the phages 1/5th volume of PEG/NaCl was added to the supernatant and incubated on ice for 30 min with gentle shaking. After centrifugation at 2000× *g* for 15 min at 4 °C, the supernatant was discarded, and the pellets were resuspended in PBS.

The precipitation procedure was repeated twice.

NaCl/PEG-precipitated phages were blocked with 5% BSA in PBS and incubated for 1 h. Phages that bound to streptavidin were deselected before bio-panning against biotinylated fondaparinux (bFP). They were captured by streptavidin-coupled Dynabeads (Thermo Fisher), washed, and eluted in 100 nM triethylamine. bFP was added to the depleted phages at equimolar concentrations. After incubation for 2 h, the antigen-incubated phages were added to the aliquot of selection beads for coupling. After 30 min, the beads were collected, and the supernatant was discarded. The beads were washed ten times with PBST and three times with PBS. For elution of the bound phages, 0.2 M glycine containing 0.1% BSA was added and shaken for 10 min. Eluted phages were neutralized using 1 M Tris-HCl pH 8.0 and propagated in TG1 *E. coli.* M13KO7 helper phages were added at a 20-fold multiplicity factor to the infected *E. coli* and induced phage production for subsequent selection rounds. Several rounds of panning and infection were performed, which finally yielded a polyclonal mixture of phages displaying proteins enriched for antigen binding [76,77]. After three rounds of selection, the output phage titers were around 1.8 × 10^10^ CFU. They did not increase drastically, indicating that the phages were sufficiently enriched and no further selection rounds were necessary.

#### 4.2.6. Monoclonal Phage ELISA

Infected *E. coli* were plated on 2×TY medium supplemented with 2% glucose, 100 µg/mL Ampicillin Agar plate, and grown overnight at 30 °C. Single colonies were picked from the Agar plates and transferred to 2×TY media supplemented with 2% glucose and 5 µg/mL tetracycline in 96 well deepwell plates. Cells were grown at 37 °C and shaken at 250 rpm until turbidity was visible. M13K07 helper phage (New England Biolab, MA, USA) was added with a 20-fold multiplicity factor, incubated at 37 °C, 50 rpm shaking for 30 min, and centrifuged at 2500× *g* at 4 °C for 10 min. The supernatants were discarded from the wells, and 2×TY media supplemented with 100 µg/mL ampicillin and 50 µg/mL kanamycin was added to each well and incubated overnight at 30 °C while shaking at 250 rpm.

High-binding 96 well microplates (Greiner, Austria) were coated with 0.5 μg/100 µL streptavidin per well for 4 h at RT and washed three times with PBST and once with PBS. To check the binding of these phages to other GAG species, separate plates were then coated with 20 pmol/well of biotinylated GAG antigens. Biotinylated HS, LMW Heparin, Chondroitinsulfate, and Dermatansulfate plates, and a streptavidin control were prepared and incubated overnight at 4 °C.

The deepwell plates were then centrifuged for 10 min at 2500× *g*, the supernatant of each well was transferred to a clean deepwell plate, and the phages were blocked with 10% BSA for 1 h at RT. After blocking, the ELISA plates were washed three times with PBST. Blocked phages were transferred to each of the corresponding blocked wells of the coated ELISA plates and incubated at RT static for 1 h, followed by 6 times washing with PBST. HRP-anti-M13-phage mAb (1:4000 dilution) in 1% BSA/PBST was added and incubated at RT for 1 h and RT, followed by 6 times washing with PBST. The reaction was developed with SureBlue TMB Peroxidase substrate (Thermo Fisher) and stopped with 1% HCl after 2 min. The evaluation was performed at 450 nm using a VarioSkan plate reader (Thermo Fisher).

A total of 90 clones with strong signals and low cross-reactivity were selected for DNA sequencing, which was performed by GeneWiz (Leipzig, 04158 Germany).

#### 4.2.7. Generation of VNAR-Fc Fusions

After the selection process and screening, the protein sequences of high-affinity binders were determined by DNA sequencing, and the VNAR sequences were reformatted to human IgG-Fc domains and expressed in HEK cells. Three different VNAR clones were cloned into the pFUSE expression vector and transiently transfected as VNAR human Fc fusions into HEK293 cells (ATCC, Manassas, VA, USA). After 5 days of growth, the cell cultures were centrifuged at 2000 rpm for 10 min, and the supernatants were filtered using 0.22 µm membrane filters. To purify the VNAR-Fc, the cell culture supernatant was loaded onto HiTrap MabSelect SuRe columns (Cytiva, Marlborough, MA, USA) pre-equilibrated with PBS, pH 7.4. Fc-VNARs bound to the Protein A column were eluted with 0.1 M glycine, pH 3.5, and the buffer was exchanged to PBS; pH 7.4 was carried out using a HiPrep 26/10 desalting column (Cytiva, Marlborough, MA, USA). The purity of the purified protein samples was determined using analytical SEC and SDS-PAGE. Binding to different GAG species was repeated for Fc-VNAR, as described previously.

#### 4.2.8. Surface Plasmon Resonance

To determine whether the VNAR-Fc fusions were specific fondaparinux binders and to determine their cross-reactivity with other GAG species, the Fc-VNAR-constructs were characterized by surface plasmon resonance (SPR) measurements on a Biacore X100 system (Cytiva, Marlborough MA, USA). Biotinylated FP, LMWH heparin, biotinylated HS, and biotinylated CS were immobilized on neutravidin-coated C1 sensor chips (Cytiva, Marlborough, MA, USA) as described earlier [71]. The VNAR-Fc constructs were measured at 8 concentrations starting from 10 µM with serial 1:2 dilutions in PBS with 0.005% Tween. PBS with 0.005% Tween and a total of 400 mM NaCl content to minimize non-specific ionic interactions were used as running buffer.

The association time was set to 180 and the dissociation time to 300 s. 1 M NaCl/50 mM NaOH was used to regenerate the chip after every cycle. The obtained sensorgrams were evaluated, and Kd values were determined using Biacore X100 evaluation software with default settings for affinity measurements [71].

#### 4.2.9. Fermentation and Purification of FP-Binding VNAR Fonda054-D09

Cells were grown in LB medium at 37 °C to an OD600 of 0.7. The cultures were centrifuged, and the cell pellet was resuspended in M9 (NMR) medium. After 1 h of adaption, the expression was induced by the addition of 1 mM IPTG. The expression was performed for 3 h. The cells were then harvested and frozen at −20 °C. The cell pellet was thawed and resuspended in 10× lysis buffer (20 mM sodium phosphate, 0.5 M NaCl, pH 7.4), sonicated, and centrifuged. The SN was loaded onto a HisTrap FF Crude column (GE) pre-equilibrated in binding buffer (20 mM sodium phosphate, 0.5 M NaCl, 20 mM Imidazole, pH 7.4). Elution was performed using a linear gradient to 100% elution buffer (20 mM sodium phosphate, 0.5 M NaCl, 250 mM imidazole, pH 7.4) over 20 CV. The protein-containing fractions were pooled and dialyzed against PBS. The dialyzed fraction pool was loaded onto a pre-equilibrated Fractogel EMD SO_3_^−^ column (50 mM Tris, pH 8) and eluted using a linear gradient to 100% elution buffer (50 mM Tris + 2 M NaCl, pH 8) over 10 CV. The protein-containing fractions were pooled and dialyzed against 50 mM sodium phosphate and 50 mM NaCl at pH 6.5.

#### 4.2.10. NMR Spectroscopy

All NMR spectra were recorded on a Bruker Avance III 700 MHz spectrometer (Bruker Instruments, Billerica, MA, USA) equipped with a cryogenically cooled 5 mm TCI probe using *z*-axis gradients at 25 °C. The protein sample concentration was 0.5 mM, dissolved in phosphate buffer (50 mM NaPi, 50 mM NaCl, pH 6.5). For the assignment of the backbone resonances, standard triple resonance experiments were used: HNCO [78,79], HN(CA)CO [79,80], HNCACB [81,82], HN(CO)CA [78,79], HNCA [78,79,83], HN(CA)CO [79,80], 15N HSQC [80]. For sidechain resonance assignments, we used HCCH-TOCSY [84,85,86], HCCCONH (H-detected) [87,88], and HCCCONH (C-detected) [87,88]. To reduce water signals, 13C NOESY experiments [89] were performed in 100% D2O. For that matter, the protein sample was first lyophilized and subsequently dissolved in 100% D2O to a final protein concentration of about 0.5 mM. The mixing times of the 13C-NOESY and 15N-NOESY experiments were set to 100 ms and 80 ms, respectively. The backbone and side-change resonance assignments and structure calculations were carried out with CcpNMR 2.4.1. [90] and CYANA 3.98.5 [91,92], respectively. Spectra were processed with NMRpipe [93]. NOESY peaks were manually picked and automatically assigned using CYANA (3.98.5). The experimentally determined disulfide bonds were included in these calculations. Water refinement calculations were performed according to the RECOORD [94] protocol using CNS 1.3. [95], including the distance restraints generated by the CYANA structure calculation and torsion angles derived by TALOS+ [96]. The final structure ensemble, consisting of twenty conformers, was validated by PSVS [97], and molecular images were created using PyMOL 2.6 (Delano Scientific, San Francisco, CA, USA) [98].

#### 4.2.11. Molecular Modelling

The target for docking was the above-described anti-fondaparinux VNAR NMR structure (excluding the Fc domain). The structure of the heparin pentasaccharide was used as a ligand (PDB entry 1azx. pdb) as a starting point, which was manually extracted from the AT-III complex using the YASARA molecule-building tool to obtain the fondaparinux structure. Hydrogen atoms were added to the anti-fondaparinux VNAR using the hydrogen-bonding network optimizer of WHAT IF28 to ensure correct protonation states of Asp, Glu, and His residues, and to flip Asn, Gln, and His side chains if required. Then the anti-fondaparinux VNAR was energy-minimized with a fixed backbone. Minimization and docking were performed using the YASARA NOVA force field, which was optimized by Monte Carlo moves in the force-field parameter space to obtain a deep, stable energy minimum close to the real protein structures. The pentasaccharide was placed at 144 Fibonacci grid points on a sphere around the docking target. The axis of the heparin helix was maintained parallel to the surface of the sphere and rotated in steps of 36°. From each of these starting points, the heparin oligosaccharide was moved toward the protein until the distance between the closest atoms was 5 Å. From there, an MD docking simulation was performed with an initial heparin velocity vector of 7.50 Å/ps pointing toward the protein’s center of mass, a 10.5 Å force cutoff distance, a timestep of 1 fs for bond, angle, dihedral, and planarity, and a 2-fs timestep for nonbonded electrostatic and van der Waals forces. The protein backbone was kept fixed throughout the entire procedure, but the protein side chains, entire heparin saccharide, and all degrees of freedom were unrestrained. As soon as the impact was complete (i.e., when the center of mass of the heparin molecule did not move any further), the complex was subjected to 50 steps of steepest descent and 200 steps of simulated annealing minimization (velocity vectors multiplied by 0.9 every three steps) to remove the excess potential energy stored during impact. The complex energies were calculated without a cutoff distance. The procedure was repeated for all starting grid points, and the lowest energy complex was selected for the following MD run. The stability of the modeled complex was evaluated by running a 3-ns MD simulation in an aqueous solution using the AMBER force field. For this purpose, the docked complex was placed in a rectangular box that was larger than the complex along all three axes. The box was filled with TIP3P water, and sodium ions were iteratively placed at the coordinates with the lowest electrostatic potential until the cell was neutral. MD simulation was then conducted using YASARA for 3 ns at 298 K and constant pressure. A timestep of 1 fs was chosen for the bond, angle, dihedral, and planarity forces, and a timestep of 2 fs was chosen for the intermolecular forces. The cutoff for van der Waals interactions was 7.86 Å, electrostatic forces were calculated without a cutoff using the particle mesh Ewald algorithm, and bond lengths were not restrained.

## Figures and Tables

**Table 1:** CDR1 and CDR3 amino acid sequences of the selected FP-binding VNAR clones of selection Series 1 obtained by Sanger sequencing.

Clone	CDR3	CDR1	VNAR Type (Isoform Family)
Fonda051-A01	NVYSVTHSIQGKLRAI	PYYALA	Type 4
Fonda051-A02	NVYVHRRKTPYLTKQ	VDVARA	Type 4
Fonda051-A03	NVYCVTHSLQGKLRAM	PYYAPA	Type 4
Fonda051-A05	NVYGqCCNRRRL	SNCALP	Type 2
Fonda051-A06	NVYVYDHPQYRGGFGH	RQFAPA	Type 4
Fonda051-A07	NVYSVTHSIQGKLRAI	PYYALA	Type 4
Fonda051-A08	NVYVYDHPQYRGGVGH	RQFALA	Type 4

**Table 2:** CDR1 and CDR3 amino acid sequences of the selected FP-binding VNAR clones of selection Series 2 obtained by Sanger sequencing.

Clone	CDR3	CDR1	VNAR Type
Fonda054-A01	PRLYRSSCQGSSRR	SNCALP	Type 2
Fonda054-A06	PRLYRSSCQGSSRR	SNCALP	Type 2
Fonda054-F01	PRLFRSSCqGSSRR	SNCALP	Type 2
Fonda054-G10	PRLYSSSCqGSSRR	SNCALP	Type 2
Fonda054-H05	PRVYRSSCqGSSRR	SNCALP	Type 2
Fonda054-D09	HCRRRCGDVWC	SICALS	Type 2
Fonda054-A02	NRSYVESYDIWSKLL	SNCALS	Type 2

**Table 3:** Refinement statistics of the VNAR Structure.

*Distance constraints*	
*Total*	1912 (100%)
*Intraresidue*, *|i-j| = 0*	343 (17.9%)
*Sequential*, *|i-j| = 1*	456 (23.8%)
*medium-range*, *1 < |i-j| < 5*	191 (10.0%)
*long-range*, *|i-j| >= 5*	922 (48.2%)
***Dihedral angle constraints***	
*Total*	150
***Violations***	
*RMS of distance violation/constraint*	0.02 Å
*Maximum distance violation*	0.28 Å
*RMS of dihedral angle violation/constraint*	0.07°
*Maximum dihedral angle violation*	1.20°
***Deviations from Ideal Geometry***	
*RMS deviation for bond angles*	1.6°
*RMS deviation for bond lengths*	0.019 Å
***RMSD Values***	
*Backbone*	All: 1.5 Å Ordered: 0.3 Å
*Heavy atoms*	All: 2.1 Å Ordered: 0.7 Å
***Ramachandran Plot***	
*Most favored regions*	90.5%
*Additionally allowed regions*	9.0%
*Generously allowed regions*	0.5%
*Disallowed regions*	0.0%

## Data Availability

Supporting data can be obtained from the authors upon request.

## Abbreviations

The following abbreviations are used in this manuscript:

AT-III	Antithrombin-III
b-FP	Biotinylated fondaparinux
BSA	Bovine Serum Albumin
CDR	Complementarity-determining region
CFU	Colony-forming unit
CS	Chondroitin sulfate
D2O	Deuterium oxide
DNA	Deoxyribonucleic acid
DOAJ	Directory of open access journals
DS	Dermatan sulfate
EDTA	Ethylenediaminetetraacetic acid
ELISA	Enzyme-linked Immunosorbent Assay
FDA	Food and Drug Administration
FP	Fondaparinux
GAG	Glycosaminoglycans
GD2	Tumor-associated ganglioside
H2O	Water
HP	Heparin
HPLC	High Performance Liquid Chromatography
HRP	Horseradish peroxidase
HS	Heparan Sulfate
HV	Hypervariable regions
IFT	Isothermal Fluorescence Titration
IgG	Immunglobulin G
IgNAR	Immunoglobulin new antigen receptor
Kd	Dissociation Constant
kDa	Kilodalton
LB medium	Luria Broth medium
LD	Linear dichroism
LMWH	Low-Molecular-Weight Heparin
M	Molar
m/z	Mass to Charge Ratio
MDPI	Multidisciplinary Digital Publishing Institute
MES	2-(N-Morpholino)-ethansulfon-acid
min	Minutes
mM	Milimolar
MS	Mass Spectrometry
NaCl	Sodium chloride
NaOH	Sodium Hydroxide
NaPi	Sodium Phosphate
nM	Nanomolar
NMR	Nuclear magnetic resonance
OD600	Optical Density at 600 nm
PAGE	Oligosaccharide-Polyacrylamide Gel Electrophoresis
PBS	Phosphate-buffered saline
PEG	Polyethylenglycol
pH	Potential of hydrogen
rpm	Revolutions per minute
RT	Room temperature
sdAbs	Single-domain antibodies
SDS-PAGE	Sodium dodecyl-sulfate polyacrylamide gel electrophoresis
SEC	Size Exclusion Chromatography
SN	Supernatant
SPR	Surface plasmon resonance
TLA	Three letter acronym
Tris-HCl	Trizma Hydrochlorid -hydrochlorid
Trp	Tryptophan
VHHs	Heavy-chain variable domains
VHs	Heavy chains
VNARs	Variable domain of new antigen receptor
µg/mL	Microgram per milliliter
